# Lessons from the COVID-19 Pandemic on the Use of Artificial Intelligence in Digital Radiology: The Submission of a Survey to Investigate the Opinion of Insiders

**DOI:** 10.3390/healthcare9030331

**Published:** 2021-03-15

**Authors:** Daniele Giansanti, Ivano Rossi, Lisa Monoscalco

**Affiliations:** 1 Centre Tisp, Istituto Superiore di Sanità, 00161 Roma, Italy; 2 Faculty of Medicine and Psychology, Sapienza University, Piazzale Aldo Moro, 00185 Roma, Italy; ivano.rossi.univ.sap@gmail.com; 3 Faculty of Engineering, Tor Vergata University, Via Cracovia, 00133 Roma, Italy; lisamonoscalco@hotmail.com

**Keywords:** eHealth, medical devices, mHealth, digital radiology, picture archive and communication system, artificial intelligence, electronic surveys, chest CT, chest radiography

## Abstract

The development of artificial intelligence (AI) during the COVID-19 pandemic is there for all to see, and has undoubtedly mainly concerned the activities of digital radiology. Nevertheless, the strong perception in the research and clinical application environment is that AI in radiology is like a hammer in search of a nail. Notable developments and opportunities do not seem to be combined, now, in the time of the COVID-19 pandemic, with a stable, effective, and concrete use in clinical routine; the use of AI often seems limited to use in research applications. This study considers the future perceived integration of AI with digital radiology after the COVID-19 pandemic and proposes a methodology that, by means of a wide interaction of the involved actors, allows a positioning exercise for acceptance evaluation using a general purpose electronic survey. The methodology was tested on a first category of professionals, the medical radiology technicians (MRT), and allowed to (i) collect their impressions on the issue in a structured way, and (ii) collect their suggestions and their comments in order to create a specific tool for this professional figure to be used in scientific societies. This study is useful for the stakeholders in the field, and yielded several noteworthy observations, among them (iii) the perception of great development in thoracic radiography and CT, but a loss of opportunity in integration with non-radiological technologies; (iv) the belief that it is appropriate to invest in training and infrastructure dedicated to AI; and (v) the widespread idea that AI can become a strong complementary tool to human activity. From a general point of view, the study is a clear invitation to face the last yard of AI in digital radiology, a last yard that depends a lot on the opinion and the ability to accept these technologies by the operators of digital radiology.

## 1. Introduction

As for all important diseases, for COVID-19, scholars and scientists have immediately focused on the search for a diagnostic methodology that could give an effective identification response.

Since the first studies related to the appearance of COVID-19, it has been hypothesized that radiography could represent a valid tool [1,2] in the diagnosis of COVID-19 cases. However, it was initially thought that the image alone (therefore, without the application of AI) could not be sufficient due to the possibility of confusion with other pathologies [3]. Scientists and stakeholders moved on to the reverse transcriptase-polymerase chain reaction (abbreviated RT-PCR) [4,5], which was tested and inserted as a gold standard after approval by the CDC and the WHO to identify the virus causing COVID-19. The RT-PCR allows discrimination with other beta-coronaviruses [4,5], and in the context of molecular diagnostics with an appropriate articulated laboratory set-up with certain technical times [6,7], a genomic detection of the virus [8,9].

### 1.1. Problems with the Use of RT-PCR

This gold standard is not perfect [3], as some studies have reported false negatives [9], and the process is not free from potential errors [10,11,12,13,14,15].

Furthermore, all health systems are stressed in the use of the gold standard RT-PCR for the following obvious reasons:

1. The big demand is undermining supplies that are very complex due to complex kits and materials to be found during the pandemic.
2. The type of test is particularly expensive due to both the kits and the materials used (the handling difficulties in the COVID-19 era are further increasing in price), both for man time in processing.
3. The reactions involved require important technical times. Time in the pandemic era is showing itself as an important parameter, and is strongly correlated with contact tracing. Longer time implies a higher risk of spreading the SARS-CoV-2 virus.
4. The type of test requires personnel trained in specific degree courses in biomedical laboratory techniques and/or biology.
5. The specific training referred to in the previous point must be done in the presence of others to train the staff in the use of laboratory instruments and kits, and this is very difficult in the pandemic period, since many of the internship activities have been limited and/or replaced with remote activity.
6. Focusing only on a type of test as a gold standard from the point of view of optimization and resource management is required, and an equally effective solution is needed as a backup technique.

We therefore began to seek an answer to the above critical issues by looking with interest towards other solutions. In particular, we began to carefully observe the emerging potential of the world of digital radiology and the world of digital radiology (DR), where the emerging techniques of artificial intelligence, applied to the digital imaging and based on powerful algorithms, seem to have the chance to give important answers point by point to the criticalities reported above.

This is happening in both the X-ray and CT scan sectors.

### 1.2. Possible at the Moment to Investigate Answers That AI in Digital Radiology Could Give

It is clear that a test system based on AI used in these two sectors shows the following features:

1. It has no supply-critical issues thanks to digital techniques (there are no longer the problems of plate development).
2. It has no material cost problems (for the same reasons as above). In addition, AI can greatly reduce man time with automation.
3. It has a response time that is immediate, which translates into important advantages for contact tracing.
4. It requires trained personnel, but AI automation could make a strong contribution to cost minimization.
5. It needs training, however, the training on diagnostic images can also be practiced through remote techniques thanks to the exchange of images that can also be practiced through telemedicine systems based on eHealth and mHealth.
6. It could represent an alternative and/or backup system.

The development of artificial intelligence (AI) during the COVID-19 pandemic is there for all to see, and has undoubtedly mainly concerned the activities of digital radiology. Since the beginning of the pandemic, the opportunities of AI as a diagnostic tool for COVID-19 through chest CT (CCT) and chest radiography/radiology (CR) have begun to echo.

A simple search on Pubmed with key ((artificial intelligence) AND (chest radiography)) reports 246 studies in 2020 against 131 in 2019, equal to an increase of about 88%. An in-depth analysis with research key ((artificial intelligence) AND (chest radiography) AND (COVID-19) reports that 122 of these articles are focused on or connected to COVID-19.

A search on Pubmed with key ((artificial intelligence) AND (chest CT)) reports 168 studies in 2020 against 59 in 2019, equal to an increase of about 284%. An in-depth analysis with research key ((artificial intelligence) AND (chest CT) AND (COVID-19) reports that 96 of these articles are focused on or connected to COVID-19.

It was also hoped that, through the aforementioned applications, an effective and very fast diagnostic routine and alternative to the gold standard represented by the reverse transcriptase-polymerase chain reaction (abbreviated RT-PCR) technique above reported could be found.

The applications of AI in digital radiology have been remarkable in both sectors of the CCT and CR, as highlighted in wide-ranging reviews by Alsharif et al. [16] and by Ozhain et al. [17]. This was also achieved thanks to the dissemination of large public image databases. Pham in his study reported the usefulness of these databases [18].

In particular, his research is based on three public databases of COVID-19 chest X-rays:

(1) COVID-19 Radiography Database [19],
(2) COVID-19 Chest X-ray Dataset Initiative [20], and
(3) IEEE8023/Covid Chest X-ray Dataset [21].

The first database [18,19] reports both positive and negative images of viral pneumonia. The second database [18,19,20] reports only full-blown cases of pneumonia due to COVID-19. The third database [18,19,20,21] reports positive or suspected cases of viral bacterial pneumonia or COVID-19; besides the radiographic images, it also contains CT images.

As remarked by van Ginneken [22], in this field, numerous specific dedicated architectures have shown exceptional diagnostic performance, such as the DeepCOVID-XR algorithm [23]; CAD4COVID-Xray [24]; and CV19-Net [25]. The use of three pre-trained convolutional neural networks [18], AlexNet [26], Goog-LeNet [27], and SqueezeNet [28], was shown to be successful by Pham [18]. Many more successful examples of artificial intelligence in this area can be made, although the aim of the work is clearly not to find the best application of AI.

Nevertheless, the strong perception in the research and clinical application environment is that AI in radiology is like a hammer in search of a nail [29]. Notable developments and opportunities do not seem to be combined, now, in the time of the COVID-19 pandemic, with a stable, effective, and concrete use in clinical routine; the use of AI often seems limited to use in research applications. The recurring question is how to raise AI to a more important role in digital radiology, now in a full pandemic, and later at the end of the pandemic.

## 2. Objective

We have seen that the debate on the use of artificial intelligence during the COVID-19 pandemic is now underway, especially with the focus on the application in digital radiology. We have seen that many recent studies have reported increasing interest on AI in this specific field. Whether and how digital radiology will be affected by the fabulous development achieved by AI during the pandemic is a very important aspect. Surely an important role in this as in many other areas will be played by stakeholders, in our case politicians, territorial governors, and directors of health systems. As mentioned in the editorial [30] dedicated to the special issue entitled The Artificial Intelligence in Digital Pathology and Digital Radiology: Where Are We? opened in the Healthcare (Basel) j, this is one of the classic problems of the last yard of the introduction of AI. Stakeholders have their own sensors on healthcare actors, or at least they should be. The chosen future, the last yard, will depend a lot on the opinion of the actors. An inquiry into their opinion is therefore essential.

It is therefore necessary to focus on AI applied to digital radiology through the two techniques described and to understand the opinion of the users and, in particular, the opinion of the key figures.

In fact, in a top health system, the stakeholders who must direct technological and financial resources must first of all start from the opinion of those who will materially have to work with the renewal of the current process.

In non-pandemic times, a very useful tool was that of meetings with the so-called focus group tool with associations and/or the survey tool.

In a pandemic period, it is almost impossible to develop targeted focus groups, and the survey, perhaps particularly articulated and conducted remotely electronically, can play in addition to the traditional role of collecting opinions (automatically and with the maintenance of social distancing) that of the virtual focus group as well.

The main objective of the study is therefore to:

(a) Develop an electronic survey on this topic suitable for a multitude of healthcare professionals;
(b) Submit it and collect useful suggestions to carry out a specific survey by category useful for subsequent monitoring and interactions with the related scientific companies; and
(c) Apply it to a first category of health professionals.

## 3. Methods

In line with the aim of the study, we decided to develop a survey.

Preliminarily, we have addressed the aspects of privacy and data security.

### 3.1. Privacy Issues

As the privacy is a very basic issue in submissions of the public surveys we carefully considered this issue.

The questionnaire is anonymous, and the topic did not concern clinical trials on humans, but only opinions and expressions of their thoughts. In consideration of this, it was not considered necessary to proceed with the approval procedures of the EBR.

However, in order to improve the privacy aspects, the workgroup after the suggestion of experts decided not to proceed via e-mail and to avoid requesting the municipality of residence (in small municipalities, this could lead to identification).

We therefore disseminated it capillary through social media, such as Facebook, LinkedIn, Twitter, Instagram, and Whatsapp, association sites, and, in general, using a peer-to-peer dissemination.

### 3.2. Data Protection Issues

Today, there are several electronic survey applications made available by the great IT giants, such as Microsoft and Google. In this study, Microsoft Forms was chosen, which is available in the Office 365 suite provided to the staff of the Istituto Superiore di Sanità and which for this reason respects the IT security aspects required by current regulations from a systems point of view. Therefore, the tool used for the survey was based solely on resources internal to the system and protected in compliance with the regulations, and has been used in other successful experiences [31,32,33]. Even if not necessary, since the data in the records are anonymous, the database obtained is managed with care and attention to the data and with the consequent security criteria identified by general rules of best practice in accordance with the legislation.

### 3.3. Subjects and Perspectives

Regarding the address of the survey, we turned to health personnel.

However, in consideration of the objective of this study and the survey, we also managed the survey as a focus group and developed the following reasoning.

We focused on key figures in interacting with tools and processes and exposure to the Sars-Cov-2 virus. An RT-PCR study would have considered the biomedical lab technician involved in culture preparation and process maintenance as a figure.

Our study has focused more on the figures who legally have to do with radiological processes and have, due to this role, a greater exposure with the virus in the radiology environment.

These figures are those of the medical radiology technicians.

The survey was sent to a large number of subjects, as illustrated in the results, however, the analysis, with the aim of the prospective article, focused on the figure of the medical radiology technician (MRT).

## 4. Results and Discussion

The first result is represented by the environment with the core element eS.

**Figure 1:**
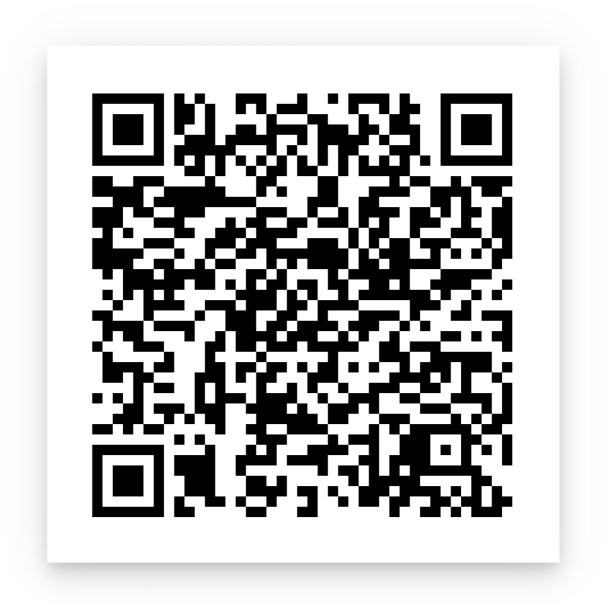
The Quick Response code of the electronic survey.

Figure 1 shows the Quick Response code related to the eS with the following link: https://forms.office.com/Pages/ResponsePage.aspx?id=DQSIkWdsW0yxEjajBLZtrQAAAAAAAAAAAAZ__gdk7kpUM1JaVENLN01ER0IwWFM0SDdHNjY4TzNKMi4u (access on 13 March 2021).

**Figure 2:**
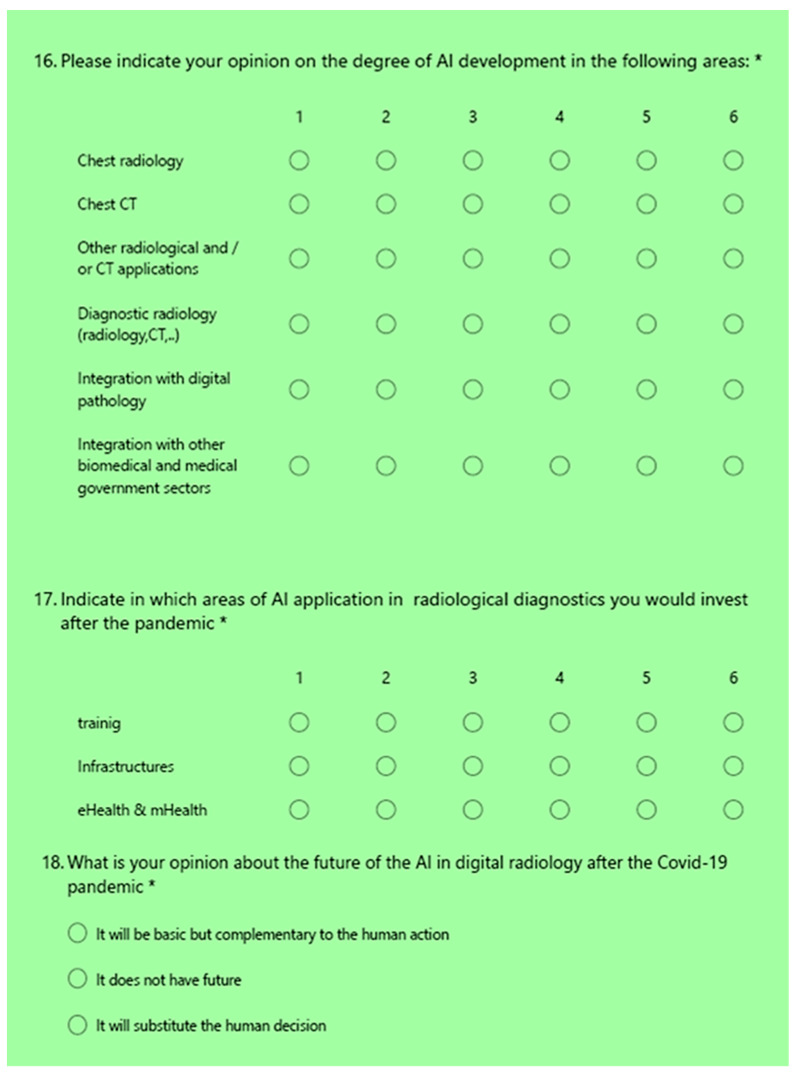
The questions focused on the perceived future of AI after the pandemic N. Q16, Q17, Q18.

Figure 2 shows the questions related to the perceived future of AI in DR after the pandemic.

### 4.1. Numerical Outcome from the Survey

The second result is the outcome from the submission of the eS. At the moment, we have submitted the survey, using the social networks, messaging tools, and other multimedia tools, to a wide sample of 1418 healthcare professionals; among them, 1348 agreed to participate. The submission now is terminated; it lasted from 10 January up to 20 January, and the data analysis will be suitably deepened by means of a specific datamining. Here, with the aim of the perspective overview of the article, we present the outcome from 182 healthcare professionals and medical radiology technicians directly focused in the interaction with the radiology infrastructure. It must also be considered that the survey was designed as a general purpose survey, and with the analysis of the results from the submission on MRTs, as well as validation of the suggestions, was intended to finalize a routine review dedicated to scientific societies.

**Figure 3:**
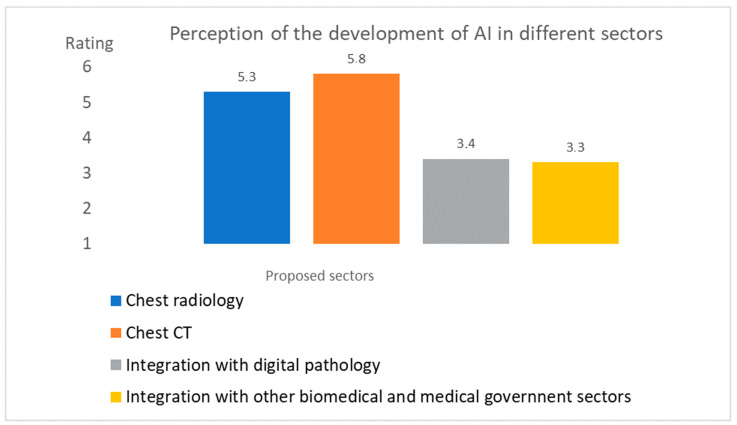
Answers to the Likert scale item #16: “Please indicate your opinion on the degree of AI development during the pandemic in the following areas”.

Figure 3 shows the answers to Likert scale item #16: “Please indicate your opinion on the degree of AI development during the pandemic in the following areas”.

**Figure 4:**
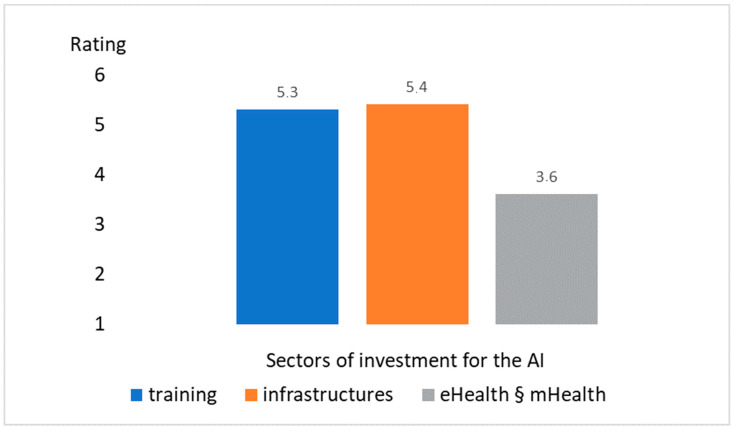
Answers to the Likert scale item #17: “Indicate in which areas of AI application in radiological diagnostics you would invest after the pandemic”.

Figure 4 shows the answers to the Likert scale item #17: “Indicate in which areas of AI application in radiological diagnostics you would invest after the pandemic”.

Each question was assigned a score from 1 (min score) to 6 (max score).

Therefore, the threshold of agreement (TA) with a proposed aspect was set at 3.5.

The outcome from the Likert scale at question #16 (Figure 2) highlighted that:

- Both chest CT and radiography were considered diagnostic areas of great development during the pandemic; both of the average values obtained were higher than TA.
- The other two areas of integration towards other non-radiological technologies were not considered areas of great development, having both obtained a value below the TA threshold.

The results related to the Likert scale at question #17 (Figure 2) highlighted that:

- Both training and infrastructure were considered areas to invest in as far as artificial intelligence is concerned. The values obtained were in fact well above the TA threshold.
- The integration into eHealth and mHealth instead showed a value equal to 3.6, just above the TA threshold.

**Figure 5:**
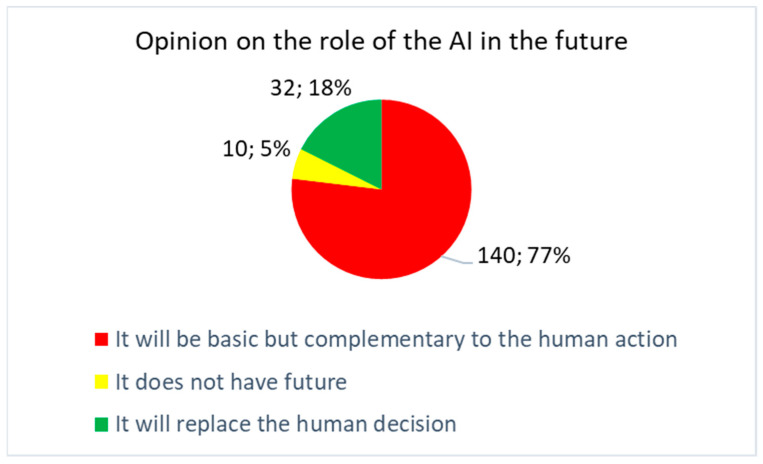
Answers to the multiple choice question #18: “What is your opinion about the future of AI in digital radiology after the COVID-19 pandemic”?

Figure 5 shows the answers to the multiple choice question #18 (Figure 2): “What is your opinion about the future of AI in digital radiology after the COVID-19 pandemic?” The result showed that, with a very high percentage of 87%, it is believed that AI will make a complementary contribution. Only 10% believed it will replace human decision. Only 3% believed it has no future.

### 4.2. Validation of the Submission on a Second Sample of MRTs

With the aim of improving and/or proving the validity of the results, we resubmitted the survey to those who did not participate in the first submission in the period from 16 to 21 February 2021 to an independent sample of 98 MRTs. Everyone joined. The repeated analysis in this sample never showed a deviation of more than 1% regarding the values illustrated in the previous analysis. The student *t*-test applied to each pair of mean values of the two submissions never showed significance in the differences between the two mean values.

### 4.3. Comments and Observations from the Survey

As it is visible through the link of the survey, it is possible to insert free comments and observations.

This is importan for collecting through the tool:

(1) Further useful information about the problem.
(2) Observations about the tool itself.

The second point is useful for preparing a further revision of the same survey that will be used several times.

Among the comments (178 in total) that emerged, we found after an interpretative synthesis:

*comm-1*  Appreciation for the initiative in various forms (150 cases), which then led to the creation of the article. In some cases, the MRT figure was particularly valued.
*comm-2*  The desire for the survey to be a stable and permanent monitoring tool (11 cases).
*comm-3*  Concern about the downsizing of one’s profession due to possible automatisms (three cases).
*comm-4*  Lack of confidence in the ability to readjust work processes (four cases) on the basis of AI.
*comm-5*  The request for further development of the survey on the needs for interaction with AI (in addition to the training one has already foreseen) (four cases).
*comm-6*  The lack of clarity of the role played in a possible process of interaction with AI (three cases).
*comm-7*  The clear separation between the world of research and the world of clinical practice in reference to the topic (two cases).
*comm-8*  The non-usefulness of the questionnaire (one case).

Some of the suggestions and observations collected directly in the survey also emerged in the peer review (see the online reports), and will be used to improve and specialize the tool in the subsequent scheduled submissions specific for the scientific societies of the MRT.

**Figure 6:**
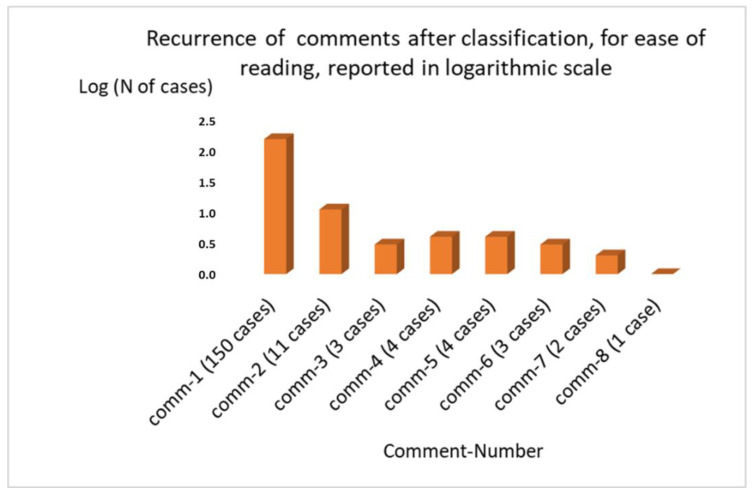
Representation in a logarithmic scale of the comments after the classification.

Figure 6 highlights in a logarithmic scale the outcome for each group of comments.

## 5. Conclusions and Work in Progress

### 5.1. Highlights in the Study

Currently, the gold standard in the diagnosis of COVID-19 identified by the CDC and the WHO is RT-PCR [3]. This test in not error free [10,11,12,13,14,15], and the process is not free from potential errors [18,19,20,21,22]. Furthermore, all health systems are stressed in the use of the gold-standard RT-PCR for the several reasons above described, ranging from the costs to difficulties in supplies. This is pushing scholars and stakeholders to look to other frontiers. The study builds on the frightening developments and the related echo of artificial intelligence in digital radiology [16,17] during the COVID-19 pandemic, and asks questions about the future developments. In particular, it (a) considers the future perceived integration of AI with digital radiology after the COVID-19 pandemic and (b) proposes a solution that, through a mechanism of electronic interaction (protected in the time of COVID-19) with professionals, makes it possible to obtain the opinions and perceptions of a key professional figure in medical radiology processes: the medical radiology technician. This solution, suitably protected regarding the aspects of privacy and data security, made it possible to automatically obtain and process such data for this figure, whose results have been evaluated and discussed, and for other figures whose datamining is continuing. A first added value is the electronic methodology, which has made it possible to prepare a survey in a structured way and in fact also acts as a virtual focus group around the MRT figure. The second added value is represented by the technological solution prepared, which is expandable, even with modifications and specialization (A) both in radiological and other non-radiological realities, such as the world of biomedical laboratory techniques, where AI is also moving, and (B) to other future periods hopefully not marked by the emergency.

The study in general and the data analysis from the survey yielded several noteworthy observations.

From the study clearly emerges the following:

- Digital radiology consists of a management process of radiological techniques and a decision-making process.
- The heart of the management process is the MRT, who interacts with the patient in the radiology laboratory and who is also the figure most exposed to COVID-19.
- Certainly, artificial intelligence could simplify processes with automation, reducing processing times (including exposure), decision times, and costs.
- Training in packages dedicated to AI applied to radiology could also be done with tutorials and remote training and exercises carried out on large public databases available, such as those shown in the study.
- The massive use of eHealth and/or mHealth solutions could make it easier to interact and finalize the further decision-making and/or administrative processes of RIS and HIS.

From the survey clearly emerges from the closed questions:

- The perception of a great development in thoracic radiography and CT, but a loss of opportunity in integration with non-radiological technologies.
- The belief that it is appropriate to invest in training and infrastructure dedicated to AI.
- The widespread idea that AI can become a strong complementary tool to human activity.

We also deepened the open questions in a dedicated final space of the survey, from which the appreciation for the initiative in various forms (which then led to the creation of the article) for the highlighting the role of the MRT figure and the desire that the tool become stable for future initiatives were most evident. In a few cases, this outcome highlights (i) the concern about the downsizing of one’s profession due to possible automatisms, and (ii) the lack of confidence in the ability to readjust work processes on the basis of AI. From this analysis (iii) some useful suggestions were also highlighted (starting from the general purpose survey) from a specialist survey focused on MRT to be used by scientific societies.

From a general point of view, the study is a clear invitation to face the last yard of AI in digital radiology, an important issue that depends a lot on the opinion and the ability to accept these technologies by the operators of digital radiology.

### 5.2. Work in Progress

From a general point of view, the questionnaire was a general purpose tool intended for a wide category of professionals. In this study, the outcome of a category of strategic professionals in digital radiology, that of MRTs, was evaluated. From this outcome and the review process, important considerations and suggestions emerged for the finalization of a specific tool for this figure to be used in scientific societies. The link below allows you to access and see some specializations prepared, and see the survey currently: https://forms.office.com/Pages/ResponsePage.aspx?id=DQSIkWdsW0yxEjajBLZtrQAAAAAAAAAAAAZ__gdk7kpUQ001Nk5ORDVPMjk0M0g4RkdPQkdOOUwwSi4u (accessed on 13 March 2021).

**Figure 7:**
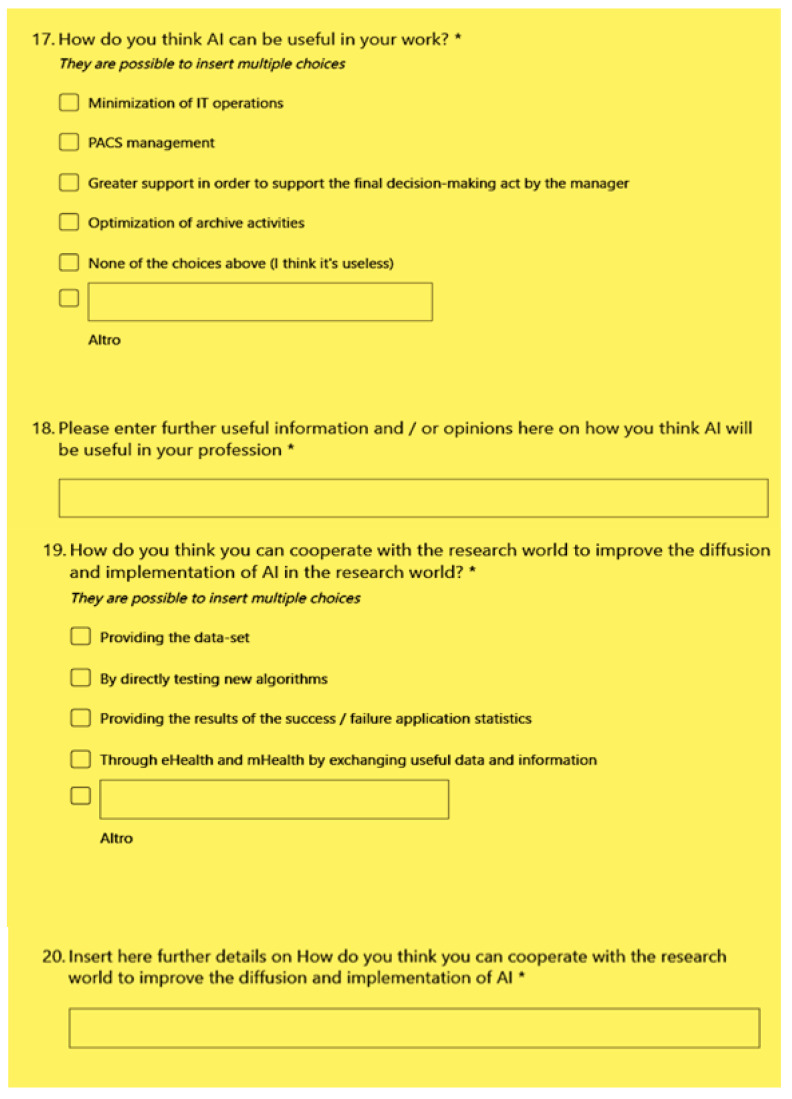
Some sections inserted in the specialized tool for the MRTs focused on the usefulness of AI in the MRT profession and the idea of cooperation of MRTs with the world of research in the field of AI.

Figure 7 shows a print screen of some changes made that allow, through two sets of multiple choice questions and two open questions, to investigate aspects of this figure’s wishes and expectations towards AI.

## Data Availability

Data sharing not applicable.
